# Temperature Sensing Performance of Microsphere Resonators

**DOI:** 10.3390/s18082515

**Published:** 2018-08-01

**Authors:** Jibo Yu, Elfed Lewis, Gilberto Brambilla, Pengfei Wang

**Affiliations:** 1 Key Laboratory of In-Fiber Integrated Optics of the Ministry of Education, College of Science, Harbin Engineering University, Harbin 150001, China; yu20131164@hrbeu.edu.cn; 2 Optical Fibre Sensors Research Centre, Department of Electronic and Computer Engineering, University of Limerick, Limerick V94 T9PX, Ireland; Elfed.Lewis@ul.ie; 3 Optoelectronics Research Centre, University of Southampton, Southampton SO17 1BJ, UK; gb2@orc.soton.ac.uk; 4 Key Laboratory of Optoelectronic Devices and Systems of Ministry of Education and Guangdong Province, College of Optoelectronic Engineering, Shenzhen University, Shenzhen 518060, China

**Keywords:** temperature sensing, microsphere resonator, material

## Abstract

In recent years, many temperature sensing devices based on microsphere resonators have emerged, attracting an increasing research interest. For the purpose of this review article, microsphere resonators are divided according to their constituting materials, namely silicone, silica, compound glass, and liquid droplet. Temperature monitoring relies mainly on the thermo-optic/thermal expansion of the microspheres and on the fluorescence of the doped ions. This article presents a comprehensive review of the current state of the art of microsphere based temperature sensing and gives an indication of future directions.

## 1. Introduction

Temperature is a vital physical parameter in industrial production, which often requires accurate temperature measurement in practical applications. It is often measured in degree Kelvin (K), which is one of the seven international standard units, degree centigrade (°C), or, less frequently in degree Fahrenheit (°F). In general, temperature is associated with many physical processes, and its measurement can be achieved using a variety of physical parameters dependent on temperature changes, thus converting temperature changes into observable physical parameters. The traditional non-optical measurement methods include volume expansion (liquid thermometer), dimensional change (bimetal thermometer), electromotive force change (the Peltier-Seebeck effect for thermocouples), and resistance change (resistance temperature detectors, light dependent resistors) [1,2,3,4,5,6,7]. Even in the case of high temperature measurements (up to 2800 °C), the temperature value has been accurately measured by employing high temperature thermocouples, bolometers, and acoustic methods [8,9,10,11].

Temperature variations are well known to have a significant influence on the optical characteristics of the materials traversed by light, thus its intensity, wavelength, phase, and polarization states [12]. In the past few years, applications of photonics in temperature sensing have been widely reported, including stand-off thermometers (pyrometers, radiation thermometers), thermometers relying on spectral changes (regenerated Fiber Bragg Gratings), and thermometers based on scattering (Raman and Rayleigh scattering) [13,14,15,16,17,18].

Optical microsphere resonators have a variety of unique advantages compared to the devices outlined above, including small size, passive photonics, lightweight, wide dynamic range, high sensitivity, and no electromagnetic interference. The principle of operation of optical microsphere resonator temperature sensors is based on a change of wavelength or intensity of a resonance peak in the reflected or output signal in response to a change of the external temperature. Details of recent works describing microsphere resonators as temperature sensors are captured in the following section.

The majority of temperature sensors based on optical microsphere resonators rely on the existence of a variable thermal expansion coefficient, which leads to a change of the refractive index or optical path length in the resonator structure, and the resulting change in wavelength or intensity of the output light signal is used for demodulation [19,20]. Conversely, in the case of microspheres whose host material is compound glass doped with rare earth ions, the temperature can be measured using the ratio of the fluorescence intensity at different wavelength bands [21,22].

In this paper, progress of temperature sensors based on the microsphere resonant cavity over the past few years is reviewed. Microsphere resonators fabricated by different materials are classified as follows:

1. Amorphous silicone microsphere resonators
2. Silica glass microsphere resonators with different structure
3. Compound glass microsphere resonators
4. Droplet microsphere resonators

## 2. Silicone Glass Microsphere Resonators

In 2009, Yang of the University of Washington proposed a microcavity resonator based on a polydimethylsiloxane (PDMS) microsphere [23]. Polydimethylsiloxane is an organic material with low loss and good chemical stability at intermediate temperatures, and is generally sensitive to temperature changes. Compared with the methods of coating organics on the surface of silica glass microspheres, the microsphere resonators based on PDMS materials were considered to be more suitable for temperature sensing [24,25]. A tapered fiber with an overall diameter of 2–10 µm was inserted into the prepared PDMS liquid, and then was drawn out rapidly, resulting in the formation of a silicone glass microsphere at the tip of the tapered fiber due to surface tension. Microspheres with different diameters were successfully fabricated using this method by varying the diameter of the tapered fiber and adjusting the viscosity of the prepared PDMS precursor monomer. 

**Figure 1:**
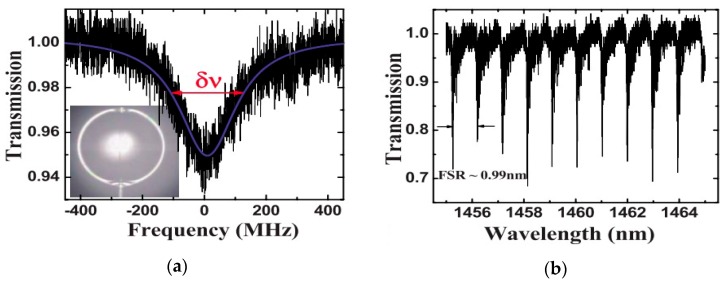
(**a**) Transmission spectra of the whispering gallery mode (WGM) resonance at *λ* = 1446.7 nm; (**b**) transmission spectrum of the polydimethylsiloxane (PDMS) microsphere in the 1460 nm band. [Reprinted/Adapted] with permission from ref [23], [AIP].

A narrow linewidth (<300 kHz) tunable laser with a wavelength of *λ* ~ 1460 nm was coupled into the silicone microsphere (of diameter 480 µm), and the transmission spectrum of the resulting whispering gallery mode (WGM) resonance is shown in Figure 1. Figure 1b shows that the free spectra range (FSR) value of the WGM is 0.99 nm, and the results of calculations show that the Q value of the PDMS microsphere was up to 10^6^ at *λ* = 1446.7 nm.

The effects of thermo-optic and thermal expansion of the amorphous silicone microsphere were calculated [23], according to Equation (1).

(1) $$\Delta \lambda =\lambda_{0}\left(\frac{1}{n}\frac{dn}{dT}\Delta T+\frac{1}{D}\frac{dD}{dT}\Delta T\right)$$

where the *λ*_0_ is the resonant wavelength of the microsphere resonator, *n* is refractive index of the silicone glass, *T* is the environment temperature, and *D* is the diameter of the microsphere. The refractive index of silicone was modulated by the thermo-optic effect, which resulted in a blue shift of the resonant wavelength, and the characteristic response time of the thermo-optic effect was several tens of microseconds [26]. On the other hand, the physical size (volume) of the microsphere resonator was changed due to the thermal expansion effect, which resulted in a red shift of the resonant wavelength, but the response time of the thermal expansion is known to be several tens of milliseconds [27].

**Figure 2:**
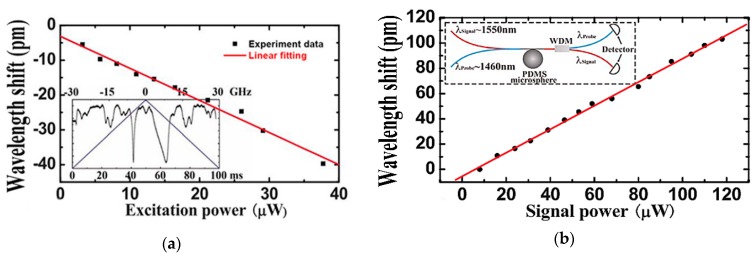
(**a**) Relationship between the wavelength shift and the excitation power in a short period of time (<10 ms); (**b**) resonant wavelength shift of the probe WGM (*λ*_0_ = 1460 nm) vs the signal power. [Reprinted/Adapted] with permission from ref [23], [AIP].

The different influence of the thermo-optic effect and of the thermal expansion were highlighted using three contrasting experiments [23]. In the first experiment, the influence of the thermo-optic effect was studied in isolation over a relatively short period of time (<10 ms); Figure 2a shows that a significant blue shift in the resonant wavelength occurred during this time interval. In the second experiment, two different wavelengths of light were coupled into the microsphere. One was a relatively weak probe light, using a tunable laser source with *λ* ~ 1460 nm and the other signal was from a laser source of *λ* ~ 1550 nm. The resonant wavelength shift was affected by the thermo-optic and thermal expansion effects simultaneously in the microsphere, and it was achieved by increasing the power of the light signal. However, in this case the thermal expansion effect played a dominant role compared with the thermo-optic effect, resulting in a red shift of the resonant wavelength, as shown in Figure 2b.

**Figure 3:**
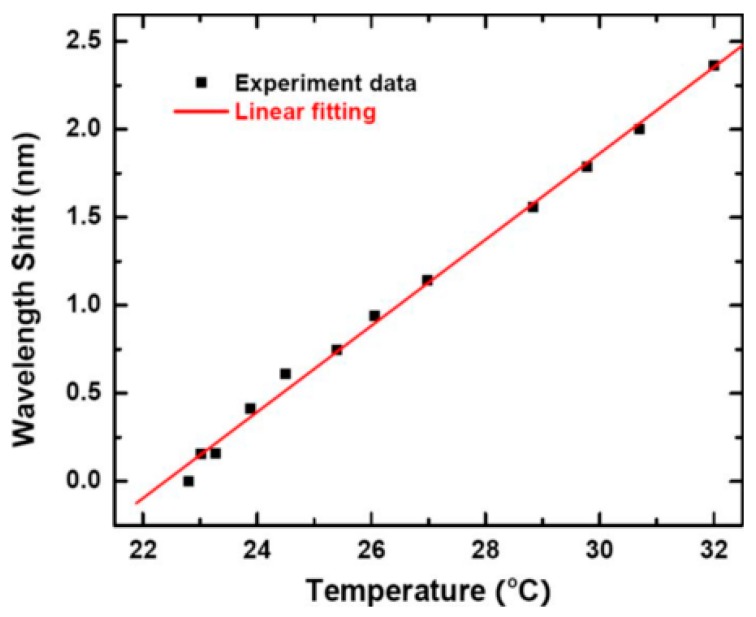
Relationship between temperature and the wavelength shift in the amorphous silicone microsphere resonator. [Reprinted/Adapted] with permission from ref [23], [AIP].

In the third experiment, only the influence of thermal expansion was considered. Figure 3 shows that when the room temperature was increased from 22 °C to 32 °C, a linear relationship was observed between the wavelength shift and temperature, resulting in a temperature sensitivity of 0.245 nm/°C, close to the theoretical value of 0.285 nm/°C calculated from Equation (1). The resolution of the detection system was considered as 0.05 pm, resulting in a temperature measurement resolution of 2 × 10^−4^ °C based on the following equation:

(2) $$\Delta T_{\min}=\Delta \lambda_{\min}/\left(d\lambda /dT\right)$$

where the Δ*λ*_min_ is the wavelength resolution of the interrogating system, dλ/dT is the temperature sensitivity of the microsphere resonator, and Δ*T*_min_ is the temperature resolution of the microsphere resonator.

## 3. Silica Glass Microsphere Resonators 

### 3.1. Conventional Silica Glass Microsphere Resonators

Silica glass microspheres have characteristics of a small dispersion value, high Q, as well as excellent chemical stability and mechanical properties such as strength, elasticity, and hardness. A simple temperature sensing device based on a silica microsphere was designed and reported in the literature [28], which placed the whole sensing device inside a copper tube to avoid the influence of external environment parameters on the temperature measurement. 

In this experiment, a pump laser with *λ* ~ 1531 nm was coupled into a silica glass microsphere through a tapered fiber, and resulted in an output spectrum at the end of the tapered fiber whose intensity was measured using a photodiode detector. By varying the diameter of the fabricated silica microspheres, a relationship between the WGM shift and the temperature changes (100 K–300 K) was obtained. It shows that the sensitivity of the silica microsphere resonator changes from 4.5 pm/K to 11 pm/K over the temperature range of 100–300 K, and the theoretical value of the sensitivity was calculated using the following equation:

(3) dλdT=(α+β)λ

where *α* is the thermal expansion coefficient and *β* the thermo-optic coefficient. According to calculations [28], the theoretical value of the sensitivity is 10.82 pm/K and 5.877 pm/K at the temperature 300 K and 150 K, respectively. Furthermore, the temperature resolution of the silica microsphere resonator was found to be 1.4 mK and 2.7 mK at the temperature of 300 K and 150 K by using Equation (2).

### 3.2. Packaged Silica Glass Microsphere Resonators

There are some differences between conventional free-standing silica glass microspheres and packaged microspheres, usually achieved using UV (ultra-violet) light to set glue with lower refractive index than silica. The packaged microsphere system could keep the coupling set-up more stable (effectively hermetically sealed), which is desirable in many practical applications. Moreover, the thermal expansion coefficient of the UV glue was approximately −3 × 10^−4^ °C, which was several orders of magnitude larger than the conventional silica glass (7 × 10^−7^ °C) [29], providing a great improvement in the temperature sensing capability. Although the Q value of the microsphere resonator decreased following packaging, it had little effect on the temperature measurement [30].

**Figure 4:**
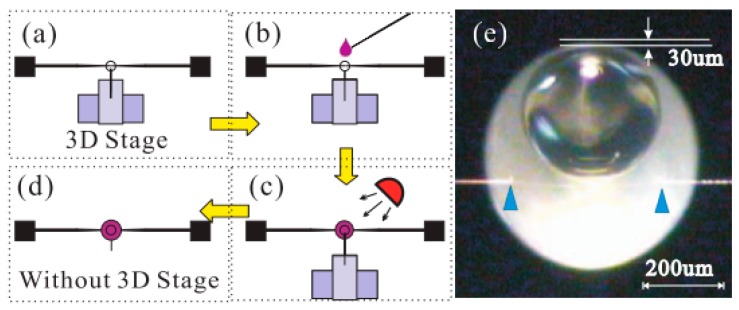
(**a**–**d**) Schematic diagram of the packaging process of the silica microsphere; (**e**) Microscope picture of the packaged microsphere aligned to the taper. [Reprinted/Adapted] with permission from ref [30], [OSA].

In this section, two types of packaged silica glass microspheres are introduced [29,30]. The first utilizes a coupling system between a tapered fiber and the silica microsphere, and the whole device was encapsulated using UV glue, as shown in Figure 4.

**Figure 5:**
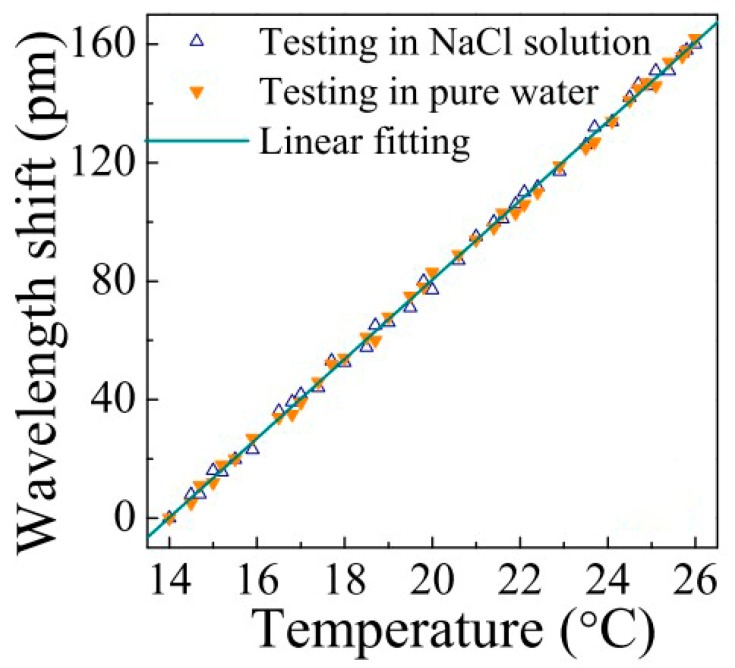
Relationship between the wavelength shift and temperature for different external solutions. [Reprinted/Adapted] with permission from ref [30], [OSA].

In this experiment, to verify the stability of the whole packaged system under the different external refractive indices, the temperature response of the wavelength shift was measured by immersing the coupling system of the packaged silica glass microsphere in a saturated NaCl (salt) solution and water. When the temperature was increased from 14 °C to 26 °C, the wavelength of the resonant peak shifted (red shift with increasing temperature) and the linear relationship is shown in Figure 5. The red shift of the WGM resonance was approximately 160.39 pm, and the wavelength shift was not affected by the external refractive index. The sensitivity of the packaged silica microsphere was determined as 13.37 pm/°C. Considering that the spectral resolution of the detector was 0.015 pm, the temperature resolution of the entire device was calculated as 1.1 × 10^−3^ K using Equation (2).

Another structure comprised an “add-drop” filter based on the coupling of a packaged silica glass microsphere, which was formed using two tapered fibers and a silica microsphere [29]. The add-drop filter has a wide range of applications in dense wavelength division multiplexing (DWDM), sensors, ultra-small optical filters, and integrated microcavity lasers. They are also effective temperature sensors and their fabrication is relatively simple with a low material cost.

**Figure 6:**
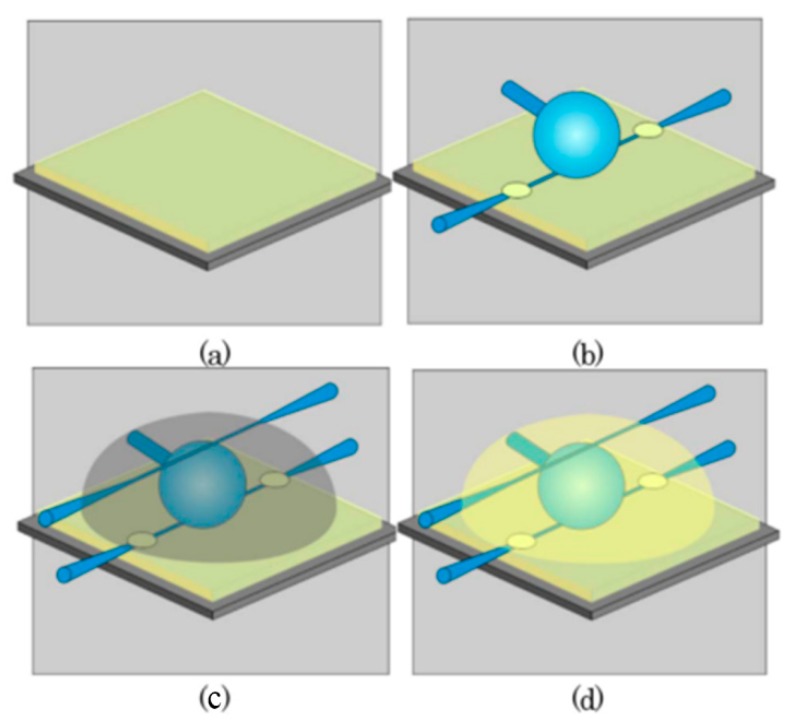
The fabrication and packaging process of the add-drop filter: (**a**) UV polymer was deposited on substrate; (**b**) the tapered fiber and silica microsphere were fixed; (**c**) the entire device were embedded in polymer; (**d**) cured with UV glue. [Reprinted/Adapted] with permission from ref [29], [OSA].

In this experiment, the UV polymer was used to cure the whole coupling device, and the packaging process is shown in Figure 6.

**Figure 7:**
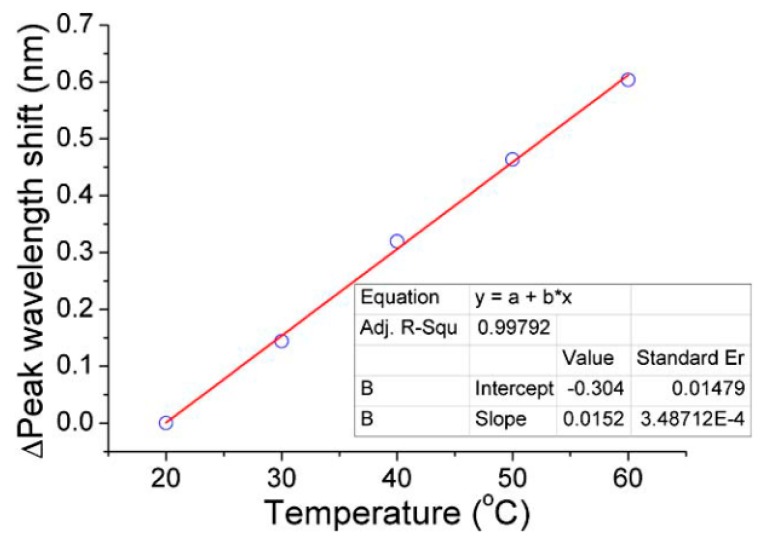
Relationship between the resonance wavelength shift and temperature change. [Reprinted/Adapted] with permission from ref [29], [OSA].

The relationship between the resulting resonant wavelength shift and temperature was obtained by changing the temperature outside of the entire coupling device, as shown in Figure 7. The wavelength shift is 604 pm when the room temperature was changed from 20 °C to 60 °C, while the resulting average temperature sensitivity was calculated as 15.2 pm/°C.

## 4. Compound Glass Microsphere Resonators

### 4.1. Nd^3+^ Doped BaTiO_3_ Glass Microsphere Resonators

Over the past few decades, there have been many reports on temperature sensing devices based on fluorescence intensity ratio (FIR) [31,32,33,34]. In this section, a Nd^3+^ doped BaTiO_3_ glass microsphere is considered [22], which has many advantages including high refractive index, high softening temperature, and high rare earth ions doping concentrations. Additionally, the temperature sensing process utilizes the FIR technique which is simpler than that used with the silica microspheres, as it does not require the use of any waveguide to couple light into the microsphere, and it can be detected at a remote distance from the doped microsphere. 

**Figure 8:**
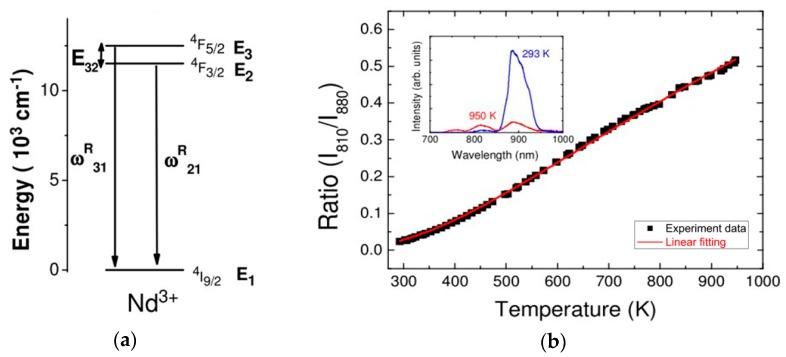
(**a**) Energy level diagram of the Nd^3+^ ions; (**b**) relationship between the fluorescence ratio and temperature. [Reprinted/Adapted] with permission from ref [22], [OSA].

Figure 8a shows the energy diagram for Nd^3+^ ions. A continuous wave (CW) light from a laser diode source with a *λ* ~ 532 nm was used to pump the Nd^3+^ ions of the doped microsphere, resulting in the transition from the *E*_1_ to *E*_2_ and *E*_3_ energy levels in the Nd^3+^ ions. Because the population of each energy level satisfied the Boltzmann distribution equation, and the probability of spontaneous emission of each energy level was different, it was concluded that the FIR (*R*) obeys the following equation:

(4) $$R=\frac{I_{31}}{I_{21}}=\frac{\omega_{31}^{R}g_{3}hv_{3}}{\omega_{21}^{R}g_{2}hv_{2}}\exp\left(\frac{-E_{32}}{KT}\right)$$

where the *E*_32_ is energy gap between the *E*_3_ and *E*_2_, $\omega_{31}^{R}$ and $\omega_{21}^{R}$ are the spontaneous emission rates of *E*_3_ and *E*_2_, $g_{2}$ and $g_{3}$ are the degeneracies of each level, and K is the Boltzmann constant. The fluorescence emission of *I*_31_ and *I*_21_ are centered around *λ* ~ 810 nm and 880 nm, respectively. The relationship of the FIR versus temperature was calculated based on Equation (4) and the result of these is shown in Figure 8b, clearly showing that the ratio increased with increasing temperature.

**Figure 9:**
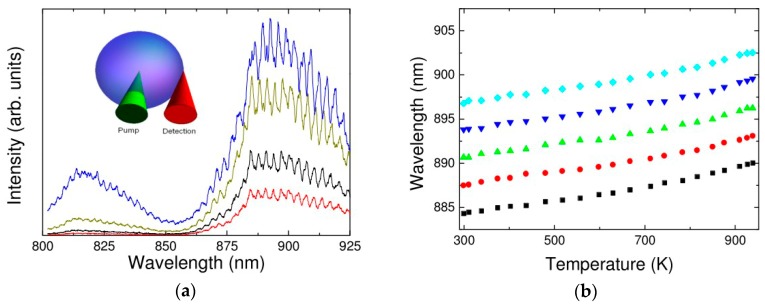
(**a**) Fluorescence spectrum of the Nd^3+^ doped microsphere; (**b**) relationship between the wavelength of the WGM resonance and temperature. [Reprinted/Adapted] with permission from ref. [22], [OSA].

The pump light was directly incident into the center of the doped ions microsphere, and a detection pinhole was placed on the surface between the microsphere and its air boundary to collect the fluorescence emission. The resulting output fluorescence spectra at different pump powers are shown in Figure 9a. The inset shows the pump and detection diagram of the microsphere.

When the environment temperature was increased from 300 K to 950 K, the resulting relationship between the temperature and wavelength for each WGM resonance was obtained, as shown in Figure 9b, from which it was calculated that the average sensitivity of Nd^3+^ doped glass microsphere was 10 pm/K.

In Reference [22], the temperature resolution of FIR and fluorescence WGM technique were evaluated. Firstly, according to Equations (1) and (3), the temperature sensitivity of FIR and fluorescence WGM can be concluded as follows:

(5) $$S_{FIR}=\frac{\delta R}{\delta T}=\frac{E_{32}}{kT^{2}}R_{0}$$

(6) $$S_{WGM}=\frac{\delta \lambda }{\delta T}=\left(\frac{1}{n}\frac{\delta n}{\delta T}+\frac{1}{r}\frac{\delta r}{\delta T}\right)\lambda_{0}$$

where *E*_32_ is the energy gap between the *E*_3_ and *E*_2_, *k* is the Boltzmann constant, *R*_0_ is the radius of the microsphere, and *λ*_0_ is the wavelength of the pumping light. Secondly, the following equation was used to calculate the temperature resolution:

(7) $$\Delta T_{\min}=\frac{{\Delta \mathrm{MP}}_{\min}}{\mathrm{MP}s}$$

where the MP*s* is the sensitivity of the measured parameter, and ${\Delta \mathrm{MP}}_{\min}$ is the limitation of measuring instrument. The MP*s* could be replaced with the sensitivity of the FIR and fluorescence WGM techniques. Finally, the resulting temperature resolution of the FIR and of the fluorescence WGM technique were determined to be 1 K and 0.1 K, respectively.

### 4.2. Er^3+^-Yb^3+^ Co-Doped Strontium Barium Niobate Glass Microsphere Resonators

In order to further optimize the host material and doping ion concentration of the compound glass, glass microspheres based on Er^3+^-Yb^3+^ co-doped strontium barium niobate (SBN) have been successfully fabricated [35]. Due to the large absorption cross-section of the SBN glass, the energy in the Yb^3+^ ions could be transferred to the adjacent Er^3+^ ions effectively, resulting in a green light emission [36].

**Figure 10:**
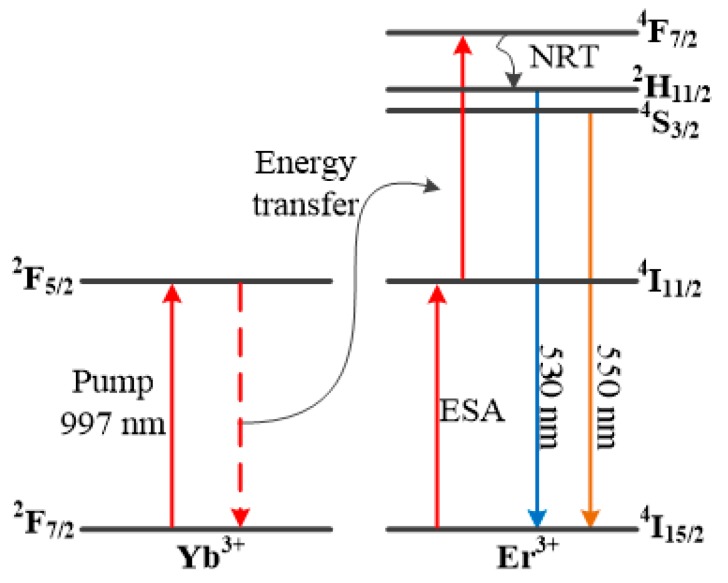
Energy level diagram of Er^3+^ and Yb^3+^ ions.

The temperature sensing capability of Er^3+^-Yb^3+^ co-doped SBN glass microsphere was found to be similar to that of the compound glass microsphere introduced in Section 4.1, both of which exploited temperature monitoring using FIR and fluorescence WGM techniques. The major difference was in the experimental measurement principle. In the case of the SBN glass microsphere dual fluorescence generation based on the upconversion between Er^3+^ and Yb^3+^ ions was utilized, and the energy level diagram is shown in Figure 10. The electrons of the Yb^3+^ ions were initially excited from the ground state ^2^F_7/2_ to the high order energy level ^2^F_5/2_ when exposed to a laser diode with *λ* ~ 997 nm. When the electrons of high order level in Yb^3+^ ions returned to the ground state, energy transfer between Yb^3+^ and Er^3+^ ions was promoted, and the electrons of the ^4^I_11/2_ were further pumped to the ^4^F_7/2_ state. Finally, the resulting fluorescence emission was observed in the wavelength range 550 nm and 530 nm due to the effect of non-radiative transition and spontaneous emission.

### 4.3. Chalcogenide Glass Microsphere Resonators

**Figure 11:**
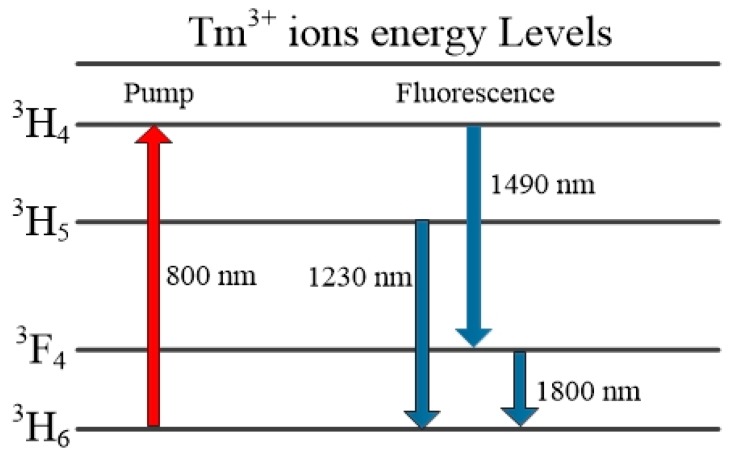
Energy level diagram of Tm^3+^ ions.

Chalcogenide glass has many advantages compared to silica and doped silica glass including high photosensitivity, low softening point, high infrared transmittance, and high sensitivity to environmental parameters such as temperature. A low temperature sensing device based on a Tm^3+^ doped chalcogenide glass microsphere has been reported [20]. A laser diode source with *λ* ~ 808 nm was used as a pump. The energy level diagram for the Tm^3+^ ions is shown in Figure 11. The Tm^3+^ ion is initially excited from the ^3^H_6_ state were to ^3^H_4_, and the resulting fluorescence emission was in the wavelength range 1.8 µm due spontaneous emission. 

In this experiment [20], the temperature was measured using the same fluorescence WGM technique presented in Section 4.1. When the environmental temperature increased from 26 °C to 97 °C, the resulting red shift of the fluorescence spectrum was 2 nm. The sensitivity of the device was measured as 28 pm/°C. This compares favorably with the theoretical value calculated as 26 pm/°C using Equation (5).

### 4.4. BaTiO_3_ Microsphere Resonators Based on Inwall Capillary

**Figure 12:**
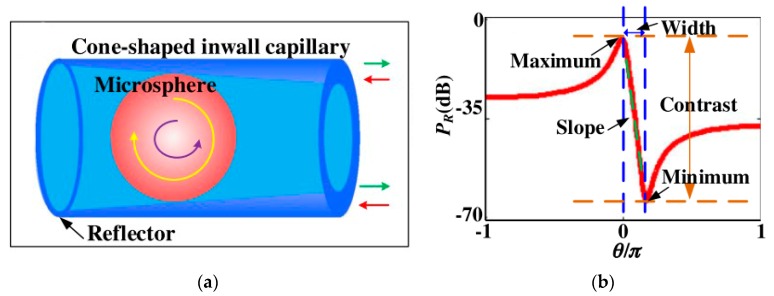
(**a**) The experimental schematic of the Fano resonator; (**b**) relationship between the Fano resonant output power and normalized frequency. [Reprinted/Adapted] with permission from ref [39], [OSA].

The profile of the Fano resonant spectra are not symmetric like the Lorentzian lines observed in conventional resonators [18]. The output spectrum changes drastically over a small frequency range and is therefore well suited for high sensitivity measurements [37,38]. A method of fabricating the Fano resonator has been reported in the literature [39]. The experimental schematic is shown in Figure 12a, in which a BaTiO_3_ glass microsphere was inserted into a prepared capillary, and then the pump source with *λ* ~ 1513 nm was coupled into the microsphere from both sides of the capillary wall. Finally, the light signals transmitted in two different directions were coupled into the microsphere, and this interaction resulted in a Fano resonance [21]. Figure 12b shows the relationship between the Fano resonant output power and normalized frequency, and the normalized frequency is equal to signal frequency divided by the sampling frequency.

**Figure 13:**
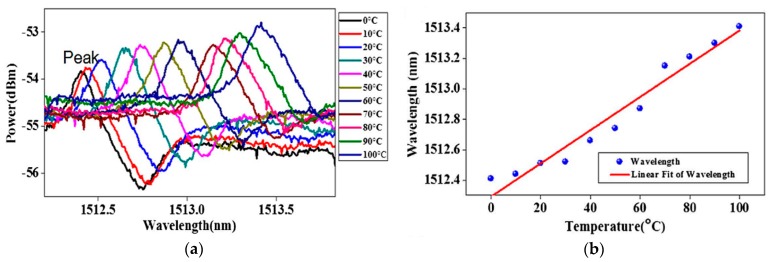
(**a**) Relationship between the Fano resonant output power and wavelength at different temperatures; (**b**) relationship between the wavelength of the Fano resonator and temperature. [Reprinted/Adapted] with permission from ref [39], [OSA].

In the temperature sensing experiment, the whole Fano resonant device was placed in a temperature chamber and the temperature increased from 0 °C to 100 °C with an interval of 10 °C. Figure 13 shows that the Fano resonant wavelength shifts and the resulting sensitivity of the device is 10.9 pm/°C. According to the Equation (6), the resulting theoretical value of sensitivity is 10.85 pm/°C and agrees well with the measured value.

## 5. Droplet Microsphere Resonators

### 5.1. Dichloromethane Dye Doped Droplet Microsphere Resonators

Most solid microspheres have some disadvantages compared with the droplet microsphere, such as low thermal expansion coefficient, non-smooth surface and large scattering, which result in a low Q value and low sensitivity of temperature sensing in microsphere resonators. Recently, a dichloromethane (DCM) droplet microsphere has been reported [19], with a high thermal expansion coefficient (>10^−3^ °C) and sensitivity to temperature changes. In this paper, an optical tweezers technique can be used to capture the droplet microspheres [40].

**Figure 14:**
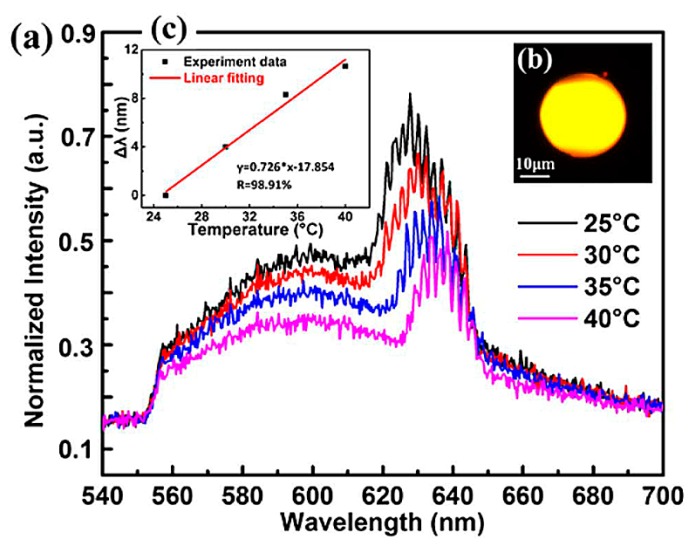
Relationship between the normalized intensity and wavelength at different temperatures. Inset: dependence of the wavelength shift on the temperature. [Reprinted/Adapted] with permission from ref [19], [OSA].

In this experiment, a pulsed laser diode with *λ* ~ 532 nm was used as the pump light source. The pulse width was 10 ns, and the repetition frequency 8 Hz. The suspended dye liquid microspheres were pumped using optical tweezers. When the diameter of the doped microsphere was 30 µm and the pulsed laser power was lower than the lasing threshold, the resulting fluorescence spectrum was as shown in Figure 14. When the environmental temperature changed from 25 °C to 40 °C, the wavelength of each resonant peak shifted, and the resulting temperature sensitivity of the droplet microsphere was measured too be 0.726 nm/°C, which is at least one order of magnitude higher than the temperature sensitivity of solid microspheres.

However, the dye doped droplet microspheres have certain disadvantages for long-term measurement due to the photobleaching phenomenon of the dye, resulting in a continuous decrease of the fluorescence intensity and a slight wavelength shift of the fluorescence WGM resonance. Therefore, for this technique the temperature sensing must be completed within a time frame several minutes or less to ensure the correctness of the measurement.

### 5.2. Liquid Crystal Microsphere Resonators

The rapid development of liquid crystal technologies has played an important role in display technology and imaging systems [41,42]. Droplet microspheres made of liquid crystal have potentially wide applications in optical sensing and microsphere lasers [43,44,45]. In this section, a temperature sensing device based on the liquid crystal microsphere is introduced [46].

**Figure 15:**
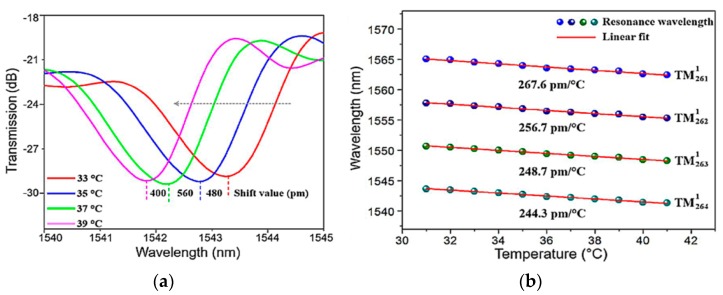
(**a**) Shift of WGM resonance at different temperatures; (**b**) relationship between the wavelength and temperature for different modes. [Reprinted/Adapted] with permission from ref [46], [OSA].

Incident light with a broadband amplified spontaneous emission (ASE) range from 1525–1570 nm was coupled into a liquid crystal microsphere using a tapered fiber, and the diameter of the microsphere was chosen to be 78 µm. The resulting WGM resonances were observed using an optical spectrum analyzer (OSA) and are shown in Figure 15a. By changing the environment temperature, a shift in the resonant wavelength could be observed from the transmission spectrum, and the resulting relationship between the wavelength and temperature is shown in Figure 15b.

Figure 15a shows that the resonance experiences a blue shift, due to the anomalous refractive index in the liquid crystal microspheres [47]. Figure 15b shows the linear fit of the WGM resonance shift, from which it was determined that the maximum sensitivity is 267.6 pm/°C when the chosen resonant mode was ${\mathrm{TM}}_{261}^{1}$. In addition, considering the resolution of the OSA as 0.02 nm, the resulting temperature resolution of the liquid crystal microsphere was calculated to be 7.5 × 10^−2^ °C using Equation (6).

## 6. Comparison between Different Types of Microsphere Resonators

A comparison between different types of microsphere resonators covered in this review article is summarized in Table 1. The temperature sensitivity of the amorphous silicone and droplet microspheres is higher than that of the glass microspheres. However, the range of temperature measurement of non-solid microspheres is far inferior to the comparable range of the solid microspheres. Therefore, a suitable microsphere resonator needs to be selected according to the specific requirements of the particular application in which it is to be used. In addition, the technique of fluorescence WGM resonance utilizes free-space coupling, which avoids the use of potentially delicate tapered fiber coupling, resulting in a more mechanically robust optical device, which is securely packaged and greater long-term stability.

## 7. Conclusions and Outlook

In this paper, a comprehensive summary of temperature sensing based on microsphere resonators has been undertaken. Microsphere resonators were classified into four types according to the different materials and signal processing methods used to observe the temperature change. Resonator devices materials reviewed included amorphous silicone, silica and compound glasses, and liquid droplets. Among them, silicone, silica, and droplet microspheres mainly access the thermal expansion and thermo-optic effects of the material to achieve temperature sensing, while compound glass microspheres use the fluorescence effect of dopant ions. In terms of temperature sensitivity, the non-solid glass microspheres are more sensitive than their solid counterparts because of their higher coefficients of thermal expansion, but the measurement range of non-solid glass microspheres is restricted. Therefore, a suitable microsphere resonator needs to be selected according to its own requirements in the desired practical application.

At present, microsphere resonators have potentially wide-ranging applications in temperature sensing. For solid glass microspheres, a feedback loop can be added to the frequency receiving device to achieve a stable frequency of the pump light, and hence reduce the thermal noise of the device by realizing ultra-stable control of the temperature. For compound glass microspheres, different rare earth ions can be introduced to further increase the sensitivity of fluorescence detection. For the liquid crystal microspheres, the internal refractive index of the microspheres can be modulated using an external electric field to obtain a transmission mode that is more sensitive to temperature changes. In general, prospects for microsphere resonators in temperature sensing are very bright and are developing rapidly.

## Figures and Tables

**Table 1:** Comparison of sensing parameters for different microsphere temperature sensors reviewed in this article.

Type	Structure	Coupling Method	Range	Maximum Sensitivity	Highest Resolution
1. Silicone glass	PDMS microsphere [23]	Tapered fiber coupling	295–305 K	285 pm/°C	0.2 mK
2. Silica glass	Silica microsphere [28]	Tapered fiber coupling	100–300 K	11 pm/°C	1.4 mK
	UV packaged silica microsphere [30]	Tapered fiber coupling	287–299 K	13.37 pm/°C	1.1 mK
	UV packaged add-drop silica microsphere [29]	Tapered fiber coupling	293–333 K	15.1 pm/°C	
3. Compound glass	Nd^3+^ doped BaTiO_3_ glass microsphere [22]	Free-space coupling	300–950 K	10 pm/°C	0.1 K
	Er^3+^-Yb^3+^ co-doped SBN glass microsphere [35]	Free-space coupling	290–380 K	4.7 pm/°C	8 mK
	Tm^3+^ doped chalcogenide glass mcirosphere [20]	Tapered fiber coupling	299–373 K	28 pm/°C	
	Cone-shaped inwall BaTiO_3_ microsphere [39]	Capillary coupling	273–373 K	10.9 pm/°C	
4. Microdroplet	DCM doped droplet mcirosphere [19]	Free-sapce coupling	298–313 K	726 pm/°C	
	LC droplet microsphere [46]	Tapered fiber coupling	306–312 K	267.6 pm/°C	75 mK
